# Variable Myopathic Presentation in a Single Family with Novel Skeletal *RYR1* Mutation

**DOI:** 10.1371/journal.pone.0069296

**Published:** 2013-07-24

**Authors:** Ruben Attali, Sharon Aharoni, Susan Treves, Ori Rokach, Michal Becker Cohen, Yakov Fellig, Rachel Straussberg, Talya Dor, Muhannad Daana, Stella Mitrani-Rosenbaum, Yoram Nevo

**Affiliations:** 1 Goldyne Savad Institute of Gene Therapy, Hadassah Hebrew University Medical Center, Jerusalem, Israel; 2 Institute of Pediatric Neurology, Schneider Children's Medical Center of Israel, Petah Tikva, Israel; 3 Departments of Anesthesia and Biomedicine, Basel University Hospital, Basel, Switzerland; 4 Department of Pathology, Hadassah Hebrew University Medical Center, Jerusalem, Israel; 5 The Unit of Neuropediatrics and Child Development, Division of Pediatrics, Hadassah Hebrew University Medical Center, Jerusalem, Israel; University of Birmingham, United Kingdom

## Abstract

We describe an autosomal recessive heterogeneous congenital myopathy in a large consanguineous family. The disease is characterized by variable severity, progressive course in 3 of 4 patients, myopathic face without ophthalmoplegia and proximal muscle weakness. Absence of cores was noted in all patients. Genome wide linkage analysis revealed a single locus on chromosome 19q13 with Zmax = 3.86 at θ = 0.0 and homozygosity of the polymorphic markers at this locus in patients. Direct sequencing of the main candidate gene within the candidate region, *RYR1*, was performed. A novel homozygous A to G nucleotide substitution (p.Y3016C) within exon 60 of the *RYR1* gene was found in patients. ARMS PCR was used to screen for the mutation in all available family members and in an additional 150 healthy individuals. This procedure confirmed sequence analysis and did not reveal the A to G mutation (p.Y3016C) in 300 chromosomes from healthy individuals. Functional analysis on EBV immortalized cell lines showed no effect of the mutation on RyR1 pharmacological activation or the content of intracellular Ca^2+^ stores. Western blot analysis demonstrated a significant reduction of the RyR1 protein in the patient’s muscle concomitant with a reduction of the DHPRα1.1 protein. This novel mutation resulting in RyR1 protein decrease causes heterogeneous clinical presentation, including slow progression course and absence of centrally localized cores on muscle biopsy. We suggest that *RYR1* related myopathy should be considered in a wide variety of clinical and pathological presentation in childhood myopathies.

## Introduction

Congenital myopathies are a heterogeneous group of neuromuscular disorders that can be subdivided, based on the predominant pathologic features observed on muscle biopsy, into: myopathies with protein accumulations, myopathies with cores, myopathies with central nuclei and myopathies with fibre size disproportion [1], [2]. While mutations in many genes may lead to similar pathological features, mutations in a single gene may also result in different muscle pathologies. Indeed, mutations in the *RYR1* gene can lead to several clinical phenotypes: Malignant hyperthermia susceptibility including King-Denborough syndrome (MHS, pharmacogenetic disorder of skeletal muscle, MIM#145600), Central Core Disease (CCD, MIM#117000), as well as some forms of Multi minicore disease (MmD, MIM#255320), Centronuclear myopathy (CNM, MIM# 160150), congenital fiber type disproportion (CFTD, MIM#255310) and other rare atypical myopathies (see [3] for review).

The ryanodine receptor 1 gene (*RYR1*) maps to chromosome 19q13.2 and is composed of 106 exons [4], [5]. It encodes one of the largest mammalian proteins (5038 amino acids, 565,176 KDa) that acts as a calcium release channel. The protein is mainly expressed in skeletal muscles where it plays a central role in excitation-contraction coupling, the process by which an electrical signal sensed by the dihydropyridine receptor (DHPR) located on the transverse tubules, is converted into a chemical signal, i.e. Ca^2+^, release from the sarcoplasmic reticulum [4].

The active channel is composed of four identical RyR1 homotetramers which assemble into a functional channel permitting the release of calcium from the sarcoplasmic reticulum stores [6], [7]. Many proteins have been reported to interact with the RyR1 (the alpha1 subunit of the DHPR, calmodulin, S100, FKBP12, triadin, homer) and to modulate its activity (see [8] for review).

So far, more than 300 mutations (source Human Gene Mutation Database, HGMD) in *RYR1* have been associated with various forms of neuromuscular disorders: MHS and CCD are mostly inherited in a dominant way whereas MmD is inherited in a recessive way. Interestingly, many dominant mutations linked to MH appear to cluster in the cytoplasmic N-terminal and central region (commonly called hot spot region 1 & 2 respectively) while mutations found in patients with CCD are predominantly localized to the C-terminal hydrophobic region (hot spot 3). On the contrary, recessive MmD mutations are spread all over the gene [9]. These observations emphasize one aspect of the complex genotype-phenotype correlations associated with *RYR1* mutations.

In the present study we describe an autosomal recessive myopathy in a large consanguineous family. The disease in this family is characterized by myopathic facial features, proximal limb muscle weakness and delay in motor milestones in all patients. However, clinical and pathological heterogeneity was noted within the family. Neonatal onset associated with no ambulation and central nuclei on muscle biopsy was found in one patient. Slowly progressive motor decline and localized fibrosis on muscle pathology in three patients resembled muscular dystrophy and were associated with delay in the diagnosis of this family. Genome wide linkage analysis revealed a single locus on chromosome 19q13. A homozygous A to G nucleotide substitution (c.9047A>G) resulting in the p.Y3016C substitution within exon 60 of the *RYR1* gene was found in the affected patients. Using the previously established method of Ca^2+^ homeostasis in EBV immortalized cells [10], [11] from affected and non affected family members, we show that the mutation we identify does not cause a shift in sensitivity of the RyR1 to activating stimuli or lead to a lower content of rapidly releasable Ca^2+^ from intracellular stores. Indeed the mutation did not seem to affect Ca^2+^ homeostasis per se, but rather lead to a decrease in protein content in muscle from the patient as determined by Western blotting. Thus the pathogenicity of the mutation is linked to protein expression which can indirectly affect excitation-contraction coupling by decreasing the amount of Ca^2+^ released because of the reduced expression of RyR1 Ca^2+^ release channels.

## Subjects/Materials and Methods

### Patients

Blood samples were obtained from affected and unaffected individuals after informed written consent. For minors/children participants, a written informed consent was obtained from their parents. DNA was extracted by using standard protocols. This study was approved by the Hadassah Ethical Review Committee.

### Pathological studies

Muscle biopsy specimens were received fresh without fixation and processed according to standard protocol. Most of the samples were snap-frozen in liquid nitrogen and cryo-preserved at −80°C. Part of the samples were fixed in formalin and embedded in a paraffin block while the remaining portions, if available, were fixed in glutaraldehyde and embedded in epoxy-resin. Paraffin sections were stained with hematoxylin and eosin (H&E) and frozen sections were stained according to standard protocols with H&E, modified Gomori-trichrome, enzyme-histochemical studies (ATPase 9.4, 4.3; NADH; SDH/COX; PAS; PAS+D; ORO) and immunohistochemical stains for fast and slow myosin. In addition, frozen sections were stained for muscle membrane proteins including spectrin, dystrophin (dys1, dys2, dys3), utrophin, sarcoglycans (alpha, beta, gamma, delta), laminin beta-1, merosin (300KD, 80KD), dysferlin, caveolin-3, perlecan, collagen IV, collagen VI and for the nuclear protein emerin, according to standard staining protocols with commercially available antibodies.

### Genome wide linkage analysis

Whole genome screening was performed using 250K SNP microarray (Affymetrix) following a standard protocol. Multipoint linkage analysis of SNP data applied to the whole genome has been performed using Alohomora [12] and Merlin softwares [13] with the following parameters: autosomal recessive inheritance, 100% penetrance and disease gene frequency in the population of 1:10 000.

### Sequence Analysis and screening of the mutation

*RYR1* gene sequencing was performed in a reference center (Laboratoire de biochimie et genetique moleculaire, CHU-Grenoble, France). All 106 coding exons were sequenced from genomic DNA from an affected individual (IV.15) and compared to the published sequences. The nomenclature was based on the reference sequence *RYR1* (GRCh37.p5 assembly, genome build 37.3, NM_000540.2), with nucleotide number 1 corresponding to the first base of the translation initiation codon.

The segregation of the mutation was checked in the pedigree by Sanger sequencing. Briefly, PCR primer pairs were designed from genomic DNA to amplify exon 60 of the isoform 1 of the *RYR1* gene including the flanking exon-intron junctions (Table 1). The purified PCR products were sequenced using the forward and reverse primers of each amplicon using standard Sanger sequencing protocol.

**Table 1:** Primers used for sequencing and mutation analyses.

	*Primer Sequence (5'-3')*	
*Primer*	*Forward*	*Reverse*
*RYR1*-Seq	acccctcattggaccctttatc	ttgcaggagacggtcagtacc
MUT	acttcaccaaccactgcctccg	ttgcaggagacggtcagtacc
WT	acttcaccaaccactgcctcca	ttgcaggagacggtcagtacc

The ARMS PCR (Amplification Refractory Mutation System) reaction was performed in 50 µl final volume with 1.6 mM MgCl_2_, 0.5 U BIOTAQ^TM^ DNA Polymerase (Bioline), 0.2 µM each primer and 50 ng DNA. Amplification parameters were: 94°C for 5 min, 30 cycles (95°C/15 sec., Tm/15 sec., 72°C/15 sec.), and 5 min. at 72°C, in an ABI 2720 Thermal Cycler (Applied Biosystem).

### Cell Culture and intracellular measurements

#### Lymphoblastoid cell lines

Mononuclear cells were isolated from peripheral blood leukocytes and transformed with EBV according to the protocol of Neitzel [14]. Cells were cultured in RPMI medium supplemented with 10 % fetal calf serum, 2 mM L-glutamine and 100 Units of penicillin and streptomycin.

#### Intracellular Ca^2+^ measurements

Changes in the intracellular calcium concentration of the lymphoblastoid cells were monitored with the fluorescent calcium indicator fura-2/AM (Invitrogen F1201) as previously described [10]. Fura-2 loaded cells (1.5×10^6^/ml; final fura-2 concentration 5 μM) were washed once by centrifugation, resuspended in Ca^2+^-free Krebs–Ringer medium containing 0.5mM EGTA and placed in a cuvette thermostated at 37°C. Fluorescence changes (ratio 340/380 nm) were measured in a spectrofluorimeter (Perkin Elmer 50B) equipped with a magnetic stirrer. All measurements were made in Ca^2+^-free Krebs–Ringer buffer containing 0.5mM EGTA (Sigma K4002). Experiments were performed at least four times on three different days.

### Western Blotting

Cryo-preserved muscle from patient V.28 was homogenized and lysed at 4°C in lysis buffer (100 mM Tris-HCl, pH 7.4, 1% Nonidet P40, 20 mM β mercapto-ethanol, 1 mM ethylene diamine tetra acetate) freshly supplemented with the protease inhibitor 1 mM phenyl-methane-sulfonyl-fluoride. The protein concentration of the lysates was determined using the Bradford quantification method. Lysates equivalent to 50 μg of total protein were fractionated by denaturing 6% polyacrylamide gel electrophoresis and transferred to nitrocellulose by standard electroblotting techniques. Western blots were probed with anti-RyR1 (Thermo Scientific mAb 34C MA3-925), anti-Ca_v_1.1 (Santa Cruz sc-8160) and anti -Myosin (Millipore MAB1628) antibodies followed by the appropriate secondary conjugate and developed using the Super Signal chemiluminescence kit (Thermo scientific 34076) as previously described [15].

### Statistical analysis

Statistical analysis was performed using Student's ***t*** test for paired samples or ANOVA when more than two groups were compared followed by the Bonferroni post hoc test. Origin computer program (Microcal Software, Inc., Northampton, MA, USA) was used for statistical analysis and dose response curve generation. Results are expressed as mean value (± SEM) of n results, where n stands for the number of measurements.

## Results

### Patients' description

Description of the patients and their family members is presented in Table 2.

**Table 2:** Clinical features and laboratory findings in the patients with myopathy.

*Patient*	*Sex*	*Current age (years)*	*Onset*	*CK*	*Weakness*	*Muscle biopsy findings*	*Motor function*	*Ocular*	*Scoliosis*
Patient 1 (IV.15)	F	30	Birth	N	Face, Neck, limb girdle	Central nuclei	wheelchair-bound	–	Y
Patient 2 (V.28)	M	17	18 months	N	Face, Neck, limb girdle	FSV, IDN, EF	Walking	–	No
Patient 3 (V.31)	M	10	6 years	N	Face, Neck, limb girdle	FSV, IDN, EF	Walking	–	Y
Patient 4 (V.36)	M	9	4 years	N	Face, Neck, limb girdle	FSV, IDN, EF	Walking	–	No

F: female; M: male; N: normal; FSV: fiber-size variation; IDN: internally-displaced nuclei; EF: endomysial fibrosis; Y:yes; No: No.

**Figure 1:**
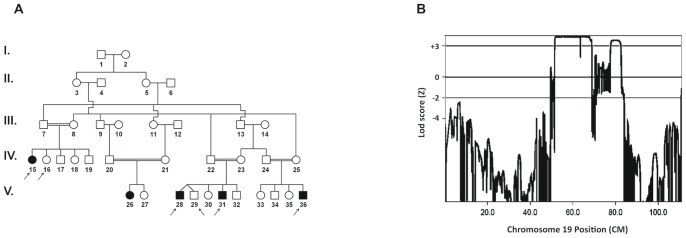
Linkage analysis of the family suffering from autosomal recessive atypical congenital myopathy. (**A**) Pedigree of the family. Filled and unfilled symbols represent affected and unaffected individuals, respectively. The arrows denote individuals whose DNA samples were analyzed by SNP250K. (**B**) Multipoint linkage analysis using SNP data showing LOD score *Z_max_* = 3.86 at θ = 0.0 on chromosome 19q13. X-axis: genetic distance in cM., Y-axis: Lod score.

**Patient 1** (IV.15) is the first child of consanguineous first cousins of Muslim origin (Fig 1A). She was born after normal pregnancy at term by vacuum delivery. Severe hypotonia was noted after birth. Gross motor development was slow. At age 6 months, the patient could not lift her head in prone position, and at the age of 18 months, she only stood with support. She never achieved independent walking. Her cognitive development was normal. Over the years, the patient had frequent hospitalizations for upper respiratory and pulmonary infections. Neurological examination at 10 years of age demonstrated decrease in muscle mass, low body weight, myopathic long face and proximal weakness. Electromyography was normal. Muscle biopsy at the age of 18 months showed variation in fiber size with central nuclei and several atrophic fibers (unfortunately the slides of this muscle biopsy are unavailable). Plasma creatine kinase levels were normal. Diagnosis at the time was centronuclear myopathy. The patient returned to our clinic at the age of 23 years. She was a university graduate. On a recent examination, her face was long and thin, with preserved facial expression. There was no limitation of extraocular movements. There was general muscle wasting and weakness most prominent in neck extensors and flexors (MRC 3/5), deltoid (MRC 3/5) and hip girdle (MRC 2/5). Tendon reflexes were elicited in the upper limbs. Her scoliosis had worsened and a CXR revealed lumbar scoliosis of 30° to the left and subluxation of the right hip joint.

**Figure 2:**
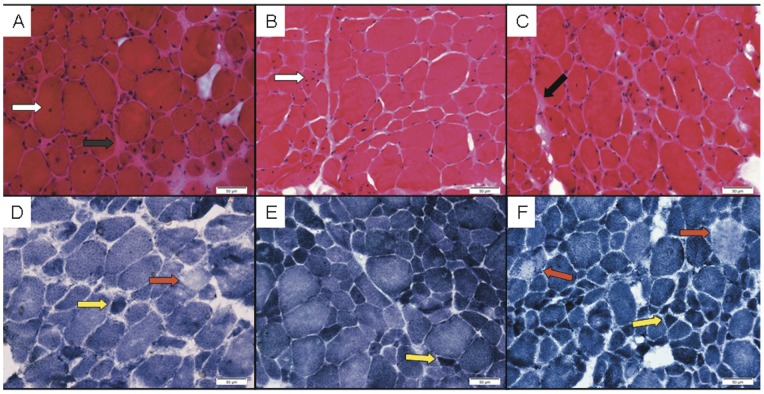
Histological analysis of patients muscle biopsies. Frozen sections of 3 cases (V28, V31 & V36) that were available for pathological review display non-specific dystrophic-like changes, consistent with muscular dystrophy. H&E stained sections (A–C) show marked variation in myofiber-diameter, in random distribution. The number of internally displaced nuclei (white arrows) is markedly increased. There are no clear-cut signs of necrosis, regeneration or any other specific structural change in the myofibers. There is focal endomysial fibrosis (black arrows). There is no inflammatory infiltrate. The blood vessels are unremarkable. NADH histochemical stain (D–F) is not showing significant changes in the cytoarchitecture, except for occasional moth-eaten-like fibers (red arrows) and overstaining of atrophic fibers (yellow fibers). (Original magnification ×40; Bars = 50 μm).

**Patient 2** (V.28) is the first cousin of patient 1 (IV.15). He was born to consanguineous cousins as part of a dizygotic twin pregnancy with uneventful delivery (Fig 1A). His mother noticed slight hypotonia and gross developmental delay compared to his twin brother. The patient walked independently at age 15 months but later could not climb stairs and required support to rise from a sitting position. He fell frequently. Intellectual performance was good. Laboratory results showed normal level of plasma creatine kinase. Electromyography revealed no abnormalities. Quadiceps muscle biopsy (Fig 2A & 2D) showed variable fiber-size distribution and an increase of internal nuclei, usually several per myofiber rather than one central nucleus. There were no clear-cut signs of necrosis, regeneration or any other specific structural change in the myofibers. There was however, focal endomysial fibrosis with no inflammatory infiltrate. These findings including focal excessive endomysial fibrosis were interpreted as suggestive of muscular dystrophy. Enzyme-histochemical studies did not reveal significant changes except for occasional moth-eaten-like fibers and overstaining of atrophic fibers on NADH stain. Semi thin sections and electron microscopy did not show any cores, minicores or any other significant change except for focal abundant fibrosis. Immunostains for muscle membrane proteins and for emerin were interpreted as normal (data not shown). Molecular testing for selenoprotein and FKRP was negative. On a recent examination at the age of 13 years, a thin long face was observed with no ptosis or ophthalmoplegia. There was general muscle wasting and weakness mainly in neck flexion and extension and hip and shoulder girdles (MRC 3+). Waddling gait and hyperlordosis were observed with positive Gower's sign. The patient is still ambulant and the parents reported slowly progressive weakness. Reduced deep tendon reflexes were elicited at the patellar tendons.

**Patient 3** (V.31) presented to our clinic at the age of 6 years because of maternal concerns about gross motor impairment, similar to what was found in his older brother (patient 2, V.28, Fig 1A). The child sat at the age of 7 months and walked independently by age 14 months with frequent falls. Perinatal history was uneventful. Physical examination demonstrated myopathic face with normal eye movements. Strength was 3+/5 in lower limb proximal muscles and 4−/5 in proximal upper limbs. Shoulder atrophy and early scoliosis were observed. Waddling gait and near positive Gowers’ sign were noted. Slowly progressive course of the disease was reported. The muscle biopsy including electron microscopy findings (Fig 2C and 2F) were similar to those described for patient number 2, except for a relative increase of type 1 fibers.

**Patient 4** (V.36) is the youngest of 4 siblings, born to consanguineous cousins. He is the first cousin of patients 1, 2 and 3 (IV.15, V.28, and V.31, Fig 1A). He was born at 32 weeks gestation via caesarian section, birth weight was 900 grams. Perinatal period was remarkable for respiratory distress syndrome which required mechanical ventilation. He was discharged from the NICU after 50 days. He achieved independent walking at 18 months of age. Gross and fine motor impairment were initially attributed to prematurity. On presentation to our clinic at the age of 4 years, neurological examination revealed myopathic face, waddling gait, and difficulty in climbing stairs. The parents reported slowly progressive weakness. Muscle biopsy findings (Fig 2B and 2E) were similar to those described for patient number 2. Electron-microscopy did not reveal core-like structures or any other specific abnormalities (data not shown). Thereafter, we were informed of yet another family member (patient V26) with similar features who was followed at another medical center.

### Genome wide linkage analysis

To identify the disease locus, genome wide linkage analysis was performed using the Affymetrix 250K SNP arrays, following standard protocol, on samples indicated by arrows (Fig 1A). Multipoint linkage analysis of SNP data applied to the whole genome revealed two loci on chromosome 19q13 with a maximum LOD score *Z_max_* = 3.86 at θ = 0.0 (Fig 1B). The candidate regions were 12.6 Mb and 2 Mb in size between SNP *rs8107011* (physical position 30,841,000) and *rs7246771* (physical position: 43,509,033) and between SNP *rs1421673* (physical position 48,757,608) and *rs4802703* (physical position 50,884,885) respectively. The study of the SNP genotype in the candidate regions allowed the restriction of the region to a single locus between SNP *rs2651101* (physical position 35,424,759) and *rs7246771* (physical position: 43,509,033). Indeed, homozygosity at each SNP locus was found only within this interval for all affected individuals of the family (available upon request). The recombinant events occurred between *rs2651101* and *rs2546019* (centromeric boundary) and between *rs2189679* and *rs7246771* (telomeric boundary). Linkage and homozygosity by descent allowed the mapping of the disease gene within this interval.

### Sequence analysis and screening of the mutation

**Figure 3:**
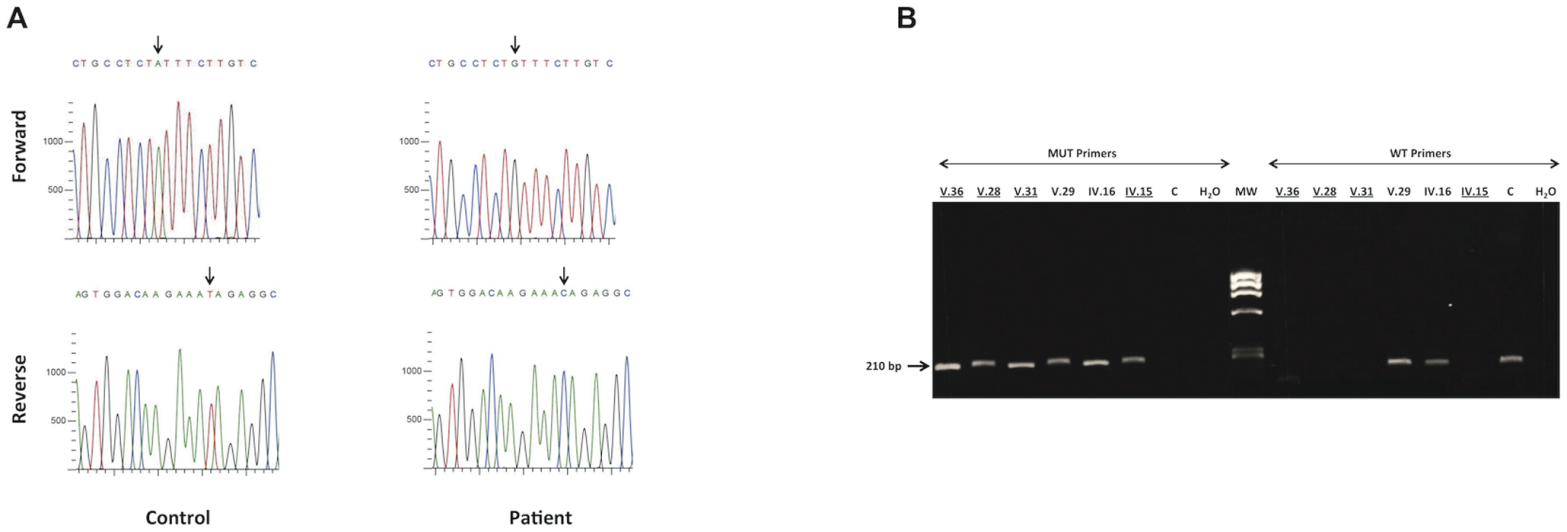
Analysis of *RYR1* at the DNA level in patients and controls. (**A**) Sequence of the *RYR1* gene revealed a homozygous A to G nucleotide substitution leading to an amino acid change (p.Y3016C) within exon 60 in patient (arrow). (**B**) Analysis of the mutation in family members and control. Left panel: PCR amplification products of *RYR1* from exon -60 to intron-60 using primers specific to the mutated allele (MUT Primers, product size = 210 bp). Right panel: PCR amplification products of *RYR1* from exon-60 to intron – 60 using primers specific to wild type allele (WT Primers, product size = 210 bp). This test confirms the cosegregation of the *RYR1* mutation with the phenotype and haplotypes in the family. C: control. Affected individuals are underlined.

Using Map viewer (NCBI), a total of 311 genes was localized in the candidate region. Among them, those expressed in skeletal muscle, or encoding proteins interacting with proteins involved in the neuromuscular system development, maintenance or diseases in either human or mouse were selected for sequence analysis. The *RYR1* gene was regarded as the best candidate as mutations within this gene are responsible for a growing number of myopathies as well as for malignant hyperthermia susceptibility [16], [17]. Because of the large size of *RYR1* gene (106 exons), mutation screening was performed in a reference center. Sequence analysis of patient (IV.15) revealed a homozygous A to G nucleotide substitution at position 9047 leading to an amino acid change, p.Y3016C (Fig 3A). All affected individuals or obligate carriers carried homozygous or heterozygous mutations, respectively, demonstrating the co-segregation of the mutation with the disease phenotype (Fig 3B). An ARMS PCR strategy was used to screen for the mutation in all available family members and in an additional 150 healthy individuals, including 50 from a North Arab Israeli population. This procedure confirmed sequence analysis and did not reveal the A to G mutation in 300 chromosomes from healthy individuals (data not shown).

### Functional properties of cells expressing the *RYR1* mutation

**Figure 4:**
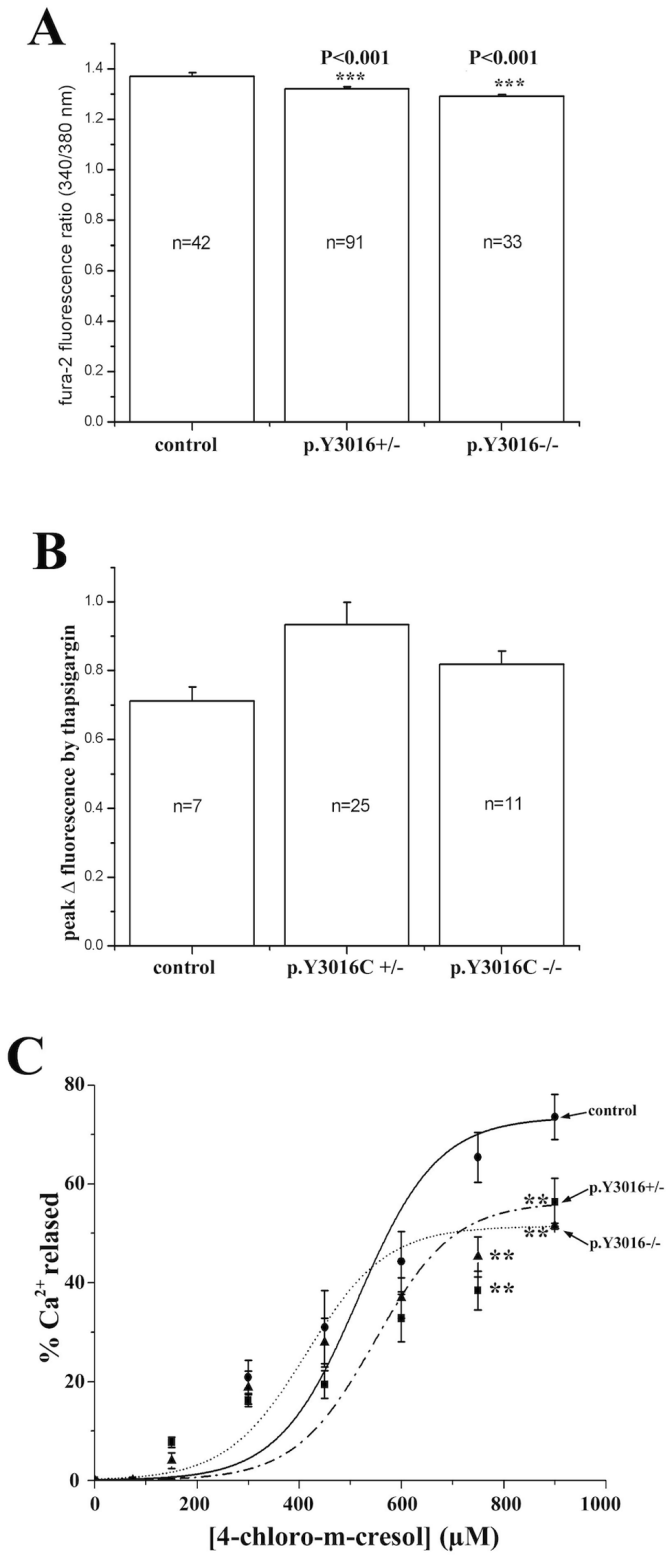
Ca^2+^ homeostasis analysis in EBV immortalized cells from affected, non affected family members and controls. (**A**) Comparison of the resting [Ca^2+^]_i_ shows lower [Ca^2+^]_i_ in cells carrying the mutation compared with control cells. **P<0.001 ANOVA followed by the Bonferroni post hoc test (**B**) The thapsigargin-induced Ca^2+^ release in lymphoblastoid B cells are represented by the difference between the resting [Ca^2+^]_i_ and the [Ca^2+^]_i_ after addition of 400 nM thapsigargin. No significant differences between cells carrying the homozygous or heterozygous p.Y3016C mutation were found. (**C**) Dose-dependent 4-chloro-m-cresol induced Ca^2+^ release in lymphoblastoid B cells. No significant changes were evident at concentrations <750 µM though at higher concentrations cells carrying the heterozygous and homozygous p.Y3016C substitution showed significantly lower Ca^2+^ release compared to control cells ***P<0.05 (ANOVA and Bonferroni post hoc test, P<0.05).

Although the RyR1 is mainly expressed in skeletal muscles, it has been previously shown that it is also expressed in EBV immortalized lymphoblastoid cells [11], [18]. Thus, in order to characterize the functional effect of the mutation, analysis was performed on lymphoblastoid cells carrying the mutation either at a homozygous or heterozygous level. Study of resting cytosolic calcium concentration showed slightly but significantly lower [Ca^2+^]_i_ in cells carrying the p.Y3016C mutation at heterozygous and homozygous states (Fig 4A). Next, the status of the intracellular calcium stores was evaluated by addition of the SERCA inhibitor thapsigargin. The addition of 400 nM thapsigargin revealed that in cells carrying the p.Y3060C +/− mutation, the ER Ca^2+^ stores were slightly larger. However, the difference did not reach statistical significance when compared to controls. No significant differences were noted in cells homozygous for the mutation (Fig 4B). Taken together, these results indicate that this mutation does not lead to leakage of Ca^2+^ from the endoplasmic reticulum calcium stores.

The sensitivity of the RyR1 to pharmacological activation was tested with the RyR1 agonist 4 chloro-m-cresol (4-cmc). At 4-cmc concentrations below 750 µM, the release of Ca^2+^ from the cells carrying the mutation (at the heterozygous and homozygous state) was similar to that of control cells (Fig 4C). Furthermore, calculation of the EC_50_ (50% effective concentration) showed no significant differences between control cells and cells carrying the mutation (p>0.05). Analysis of the complete 4-chloro-m-cresol dose dependent curve using ANOVA followed by the Bonferroni post hoc test revealed a significant decrease in 4-chloro-m-cresol Ca^2+^ release in cells carrying the heterozygous and homozygous p.Y3016C substitution only at concentrations >750 µM.

### Western blot analysis

**Figure 5:**
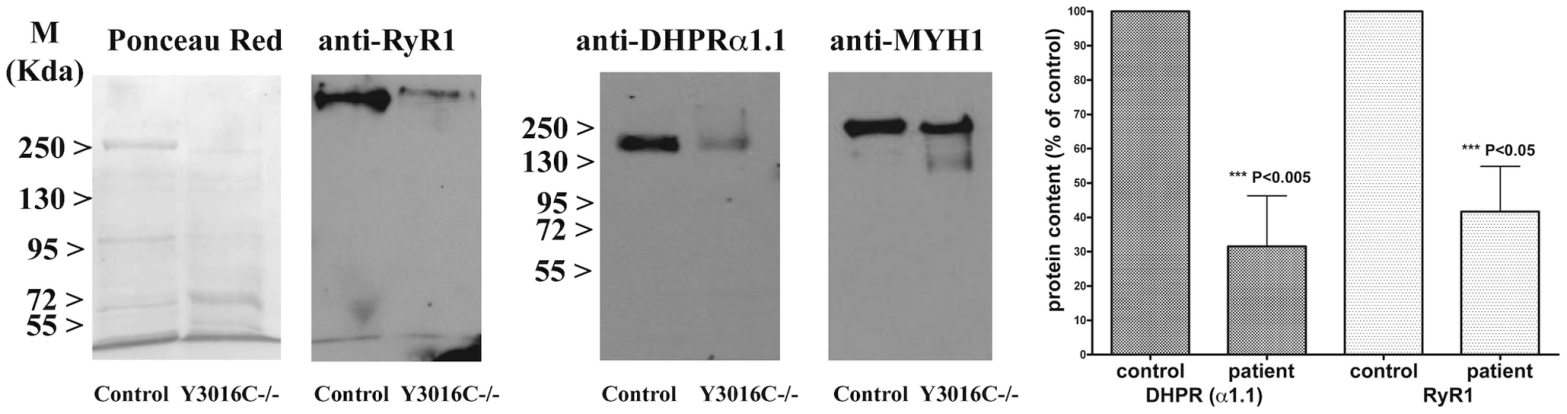
Effect of the pY3016C mutation on RyR1 protein expression on patient and control muscle biopsies. The western blot shows a dramatic decrease of the RyR1 protein and of the DHPRalpha 1.1 expression in the patient’s biopsy compared to control’s biopsy (P<0.05). Protein expression levels were quantified by densitometric analysis and normalized to the expression of myosin heavy chain. The bar plot on the right shows the mean % protein content (± SEM; of 3 different western blots) in biopsies from a control and patient Y3016C−/− (P<0.05 by the Student *t* test).

In some recessive *RYR1* myopathies, an alteration of the RyR1 protein level has been reported in the skeletal muscles [9], [19], [20]. Thus, total proteins were extracted from quadriceps muscle from patient (V.28) and control. Western Blot analysis revealed a low level of RyR1 protein (58% decrease) for the patient carrying the homozygous mutation compared with control (Fig 5). This depletion was also associated with depletion of the DHPRalpha1.1 protein (68% decrease, Fig 5). The intensity of the myosin heavy chain immunoreactive band was used as muscle specific control for the normalization of RyR1 and DHPRalpha 1.1 expression levels, attesting the veracity of results (Fig 5).

## Discussion

In the present report we performed genome wide linkage analysis of a large consanguineous family suffering from an autosomal recessive atypical and heterogeneous congenital myopathy. Our results identified a single homozygous missense mutation in the *RYR1* gene which leads to depletion of the RyR1 protein and of the DHPRα1 in muscle.

The RyR1 protein functions as the sarcoplasmic reticulum calcium release channel and also connects the sarcoplasmic reticulum and transverse tubules [4]. Mutations within this gene have been associated with several phenotypes including malignant hyperthermia susceptibility (MHS), central core disease (CCD), and multi-minicore myopathy (MmD) with external ophthalmoplegia [16], [17]. More recently, several additional atypical *RYR1*-related myopathies have been reported [3], [20]–[22].

The phenotype observed in this family was consistent with congenital myopathy regarding early onset of muscle weakness and absence of degeneration/regeneration on muscle biopsies. Furthermore, histopathological studies revealed fiber-size variation and internally-displaced nuclei which have been described in *RYR1* related centronuclear myopathies [3], [20] (CNM). Interestingly, other aspects characteristic of autosomal recessive *RYR1* related myopathies, such as the presence of cores and ocular involvement, were not present. These histopathological variabilities have been previously reported and discussed in a few cases [3], [20], [23], [24]. Specifically, Quinlivan *et al* [25] described 3 patients with dominant *RYR1* CCD where cores were absent in the youngest index case. They suggested an age effect with probable core appearance on later biopsies. The absence of ocular involvement in autosomal recessive mutations is rare but has been recently described by several clinicians [3], [20].

Several molecular mechanisms have been proposed explaining the mutations’ effect on RyR1 function (see [17], [26] for review): leaky channels and voltage sensor uncoupled channels for CCD [10], [26]–[30], hypersensitive channels for MHS [31], [32], low RyR1 expression/content for MmD [9], [33], [34], CFTD [21] and CNM [3], [20]. Interestingly, western blot analysis demonstrated a drastic reduction in the amount of RyR1 protein in these patients’ muscle biopsy. Importantly, this reduction was not paralleled by a reduced content of *RYR1* transcript as demonstrated by qRT-PCR using *MYH1* as an internal control (data not shown). However, it was accompanied by a significant reduction in DHPRalpha1 content as evidenced by western blotting. As far as functionality, using the EBV-immortalized cell model, we saw no effect of the mutation on the sensitivity of the RyR1 to pharmacological activation, though the presence of the mutation was accompanied by a slight decrease in the resting [Ca^2+^] as well as a reduced peak [Ca^2+^] release at high concentrations (>750 µM) of the RyR1 agonist 4-chloro-m-cresol. Since, the difference in 4-chloro-m-cresol-induced Ca^2+^ release was noted only for concentrations >750 µM its biological significance is difficult to explain, as is the decrease in resting [Ca^2+^] which would indicate a possible compensatory mechanism by proteins involved in lowering the resting [Ca^2+^], such as the CaATPases and /or the Na/Ca^2+^ exchanger.

Many patients with autosomal recessive *RYR1* related myopathies described in the literature have a genetic background characterized by compound heterozygous mutations, more specifically a missense mutation on one allele and a null mutation on the other [3], [19]. This could explain at least in part, the observed decrease in RyR1 protein observed in such families. However, in the present study we identified a homozygous missense mutation. The substituted residue is highly conserved among the different species and RyR1 isoforms and localized within a cytoplasmic domain which has been found to interact with apocalmodulin [35] and the II–III loop of the alpha 1 subunit of the DHPR involved in retrograde signaling [36].

It is worth mentioning that besides the p.Y3016C mutation, all affected members investigated in the present report harbor the p.G2060C polymorphism at the homozygous state. This polymorphism has been previously described as having a potential effect on the level of RyR1 protein [33]. All the members of the pedigree have been investigated for this polymorphism and individual IV.22 is homozygous for p.G2060C and healthy. This finding is important as it confirms that, when present alone, this variant is not pathogenic; indeed studies on intracellular Ca^2+^ homeostasis did not reveal any differences in resting Ca^2+^, thapsigargin-sensitive intracellular stores and sensitivity to 4-chloro-m-cresol in cells carrying the p.G2060C substitution and controls (results not shown). However we suggest that in association with other variants such as the p.Y3016C, the presence of the p.G2060C may worsen the myopathic phenotype. It would have been interesting to study the RyR1 protein level on individual IV.22 biopsy, unfortunately it was not available.

In this report we present 4 patients from an extended family with a recessive myopathy related to the p.Y3016C homozygous missense mutation in the *RYR1*. This mutation results in depletion of the RyR1 protein as well as depletion of the DHPRα1 protein. Several atypical features characterize this family. Two different phenotypes were noted in patients carrying the same homozygous mutation. One patient had neonatal onset of weakness, was never ambulatory and had central nuclei on muscle biopsy. Such presentation of centronuclear myopathy related to recessive *RYR1* mutation has been previously described [3], [20]. On the other hand, three other patients had milder initial presentation and slowly progressive muscle weakness and were still walking at the age of 17 years (patient 2), 10 years (patient 3) and 9 years (patient 4). Their muscle biopsy did not show any cores, but focal areas of fibrosis were noted. The slowly progressive course, absence of morphological sarcomere abnormalities and accumulation of fibrosis was originally interpreted as muscular dystrophy in these patients. Moreover no ocular involvement typical to recessive *RYR1* mutations was noted in any of the 4 patients.

This new *RYR1* mutation emphasizes the fact that decrease in RyR1 expression can cause heterogeneous clinical presentation in the same family, including slowly progression course without central cores on muscle biopsy. Therefore *RYR1* related myopathy should be considered in wide variety of clinical and pathological presentation in childhood myopathies.
